# *Eimeria bovis* infections induce G_1_ cell cycle arrest and a senescence-like phenotype in endothelial host cells

**DOI:** 10.1017/S0031182020002097

**Published:** 2021-03

**Authors:** Zahady D. Velásquez, Sara López-Osorio, Daniel Waiger, Carolina Manosalva, Learta Pervizaj-Oruqaj, Susanne Herold, Carlos Hermosilla, Anja Taubert

**Affiliations:** 1 Institute of Parasitology, Biomedical Research Center Seltersberg, Justus Liebig University Giessen, Giessen, Germany; 2 Research Group CIVAB, School of Veterinary Medicine, Faculty of Agrarian Sciences, University of Antioquia, Medellin, Colombia; 3 Center for Scientific Imaging, The Robert H. Smith Faculty of Agriculture, Food and Environment, Rehovot, Hebrew University of Jerusalem Israel, Rehovot, Israel; 4 Faculty of Veterinary Sciences, Institute of Pharmacology, Universidad Austral de Chile, Valdivia, Chile; 5 Cardio Pulmonary Institute (CPI), Giessen, Germany; 6 Universities Giessen & Marburg Lung Center (UGMLC), Giessen, Germany; 7 German Center for Lung Research (DZL), Giessen, Germany

**Keywords:** Apicomplexan parasites, cell cycle arrest, *Eimeria bovis*, endothelial cell, nucleolar condensation, senescence

## Abstract

Apicomplexan parasites are well-known to modulate their host cells at diverse functional levels. As such, apicomplexan-induced alteration of host cellular cell cycle was described and appeared dependent on both, parasite species and host cell type. As a striking evidence of species-specific reactions, we here show that *Eimeria bovis* drives primary bovine umbilical vein endothelial cells (BUVECs) into a senescence-like phenotype during merogony I. In line with senescence characteristics, *E. bovis* induces a phenotypic change in host cell nuclei being characterized by nucleolar fusion and heterochromatin-enriched peripheries. By fibrillarin staining we confirm nucleoli sizes to be increased and their number per nucleus to be reduced in *E. bovis*-infected BUVECs. Additionally, nuclei of *E. bovis*-infected BUVECs showed enhanced signals for HH3K9me2 as heterochromatin marker thereby indicating an infection-induced change in heterochromatin transition. Furthermore, *E. bovis*-infected BUVECs show an enhanced *β*-galactosidase activity, which is a well-known marker of senescence. Referring to cell cycle progression, protein abundance profiles in *E. bovis*-infected endothelial cells revealed an up-regulation of cyclin E1 thereby indicating a cell cycle arrest at G_1_/S transition, signifying a senescence key feature. Similarly, abundance of G_2_ phase-specific cyclin B1 was found to be downregulated at the late phase of macromeront formation. Overall, these data indicate that the slow proliferative intracellular parasite *E. bovis* drives its host endothelial cells in a senescence-like status. So far, it remains to be elucidated whether this phenomenon indeed reflects an intentionally induced mechanism to profit from host cell-derived energy and metabolites present in a non-dividing cellular status.

## Introduction

*Eimeria* spp. are widespread enteropathogens of domestic and wild vertebrates which usually cause mild pathology in affected intestine thereby resulting in either subclinical or mild clinical eimeriosis (coccidiosis). In contrast, certain bovine *Eimeria* species, such as *Eimeria bovis* and *Eimeria zuernii*, are considered as highly pathogenic, causing severe haemorrhagic typhlocolitis in calves and high-economic losses in the cattle industry worldwide (Hermosilla *et al*., 2002; Daugschies and Najdrowski, 2005). The economic impact of clinical eimeriosis has recently been attributed to 6–9% reduction of gross margin in the cattle industry (Lassen and Ostergaard 2012). As a special developmental feature, pathogenic ruminant *Eimeria* species form so-called macromeronts within host endothelial cells during first merogony which contain thousands of merozoites (*E. bovis*: >140 000 merozoites I). To guarantee this highly energy-demanding development, *E. bovis* must efficiently modulate host endothelial cells. Consistently, it has been described that *E. bovis* modulates the host cell cytoskeleton (Hermosilla *et al*., 2008*a*), inhibits host cell apoptosis (Lang *et al*., 2009), alters immunomodulatory molecule gene expression (Hermosilla *et al*., 2006; Taubert *et al*., 2006), influences nuclear factor-*κ*B activation (Alcala-Canto and Ibarra-Velarde, 2008) and exploits host cellular cholesterol metabolism (Hamid *et al*., 2014, 2015; Taubert *et al*., 2010, 2018). This overall behaviour is in line with several other intracellular parasites which are also well-known to modulate their host cell for successful intracellular development and proliferation. Besides numerous host cellular functional categories (e.g. apoptosis, autophagy, cytoskeleton, metabolism and immune reactions), several intracellular protozoa were also shown to affect the host cell cycle. Overall, parasite-triggered cell cycle perturbation was reported as both, parasite species- and host cell type-specific and to rely on a broad panel of molecular mechanisms. Thus, pathogens from different parasite classes, such as *Toxoplasma gondii*, *Leishmania* spp., *Trypanosoma cruzi* and *Encephalitozoon* spp., induce cell cycle arrest and dampen host cell proliferation (Scanlon *et al*., 2000; Kuzmenok *et al*., 2005; Brunet *et al*., 2008; Molestina *et al*., 2008; Costales *et al*., 2009; Kim *et al*., 2016), whereas *Theileria* spp. triggers host cell division and proliferation (Shiels *et al*., 2006; von Schubert *et al*., 2010; Wiens *et al*., 2014; for overview see Dobbelaere and Heussler, 1999; Dobbelaere and Rottenberg, 2003). *Leishmania amazonensis* interferes early in cell cycle by G_0_/G_1_ phase arrest (Kuzmenok *et al*., 2005) whereas *Encephalitozoon* infections induce an accumulation of host cells in the S phase (Scanlon *et al*., 2000). Besides upregulating host cellular cyclin D1, *T. cruzi* also seemed to trigger host cell progression to the S phase; however, this parasite also blocks the mitosis process and leads to impaired cytokinesis (Bouzahzah *et al*., 2008; Costales *et al*., 2009). In the case of *T. gondii*, different modes of cell cycle perturbation were reported: an infection-driven shift from G_0_/G_1_ through the S phase with the accumulation of host cells in the S phase at the G_2_/M boundary in human foreskin fibroblasts (Molestina *et al*., 2008), host cellular arrest in the G_2_ phase in a human trophoblast cell line and in human dermal fibroblasts (Brunet *et al*., 2008) or even both in an L6 rat myoblast cell line (Kim *et al*., 2016). In primary bovine umbilical vein endothelial cells (BUVECs), we recently reported on *T. gondii*-triggered G_2_/M arrest being accompanied by chromosome missegregation, the formation of supernumerary centrosomes and cytokinesis impairment (Velásquez *et al*., 2019). In contrast to *T. gondii*, a selective up-regulation of cyclin E1 was found in *Besnoitia besnoiti*-infected BUVECs thereby indicating parasite-driven host cell stasis in the G_1_ phase or at G_1_-to-S phase transition. In line, the abundance of p27-kip1, a cyclin-dependent kinase (CDK) inhibitor being regulated at the G_1_/S boundary and involved in cyclin E1–CDK2 complex activity, was also found to be up-regulated in *B. besnoiti*-infected BUVECs (Velásquez *et al*., 2020). So far, very little data exist on *Eimeria*-driven host cell cycle perturbation. In a transcriptome-based approach, *E. bovis* infection was shown to modulate gene transcription of cell cycle-related molecules, such as cyclin D2, cyclin E1, CDK2AP1, GADD45A or CDK inhibitor 1C in BUVECs (Taubert *et al*., 2010). In the case of the avian species *Eimeria tenella*, a G_0_/G_1_ arrest of HEK293T cells was induced upon transfection with an *E. tenella* rhoptry kinase 1 (Diallo *et al*., 2019).

In the current study, we show that *E. bovis* infection indeed influences cell cycle progression in infected primary bovine endothelial cells. Thus, *E. bovis* not only induces G_1_ arrest in primary bovine host endothelial cells, but also seems to drive them into premature senescence.

## Materials and methods

### Primary BUVEC isolation and maintenance

Primary BUVECs were isolated from umbilical veins obtained from calves born by *sectio caesarea* at the Justus Liebig University Giessen. Therefore, umbilical cords were maintained at 4°C in 0.9% Hanks' balanced salt solution-4-(2-hydroxyethyl)-1-piperazineethanesulphonic acid buffer (pH 7.4; Gibco, Grand Island, NY, USA) supplemented with 1% penicillin (500 U mL^−1^; Sigma, St. Louis, MO, USA) and streptomycin (500 *μ*g mL^−1^; Sigma) for a maximum of 16 h before use. For the isolation of endothelial cells, 0.025% collagenase type II (Worthington Biochemical Corporation) suspended in Puck's solution (Gibco) was infused into the lumen of ligated umbilical veins and incubated for 20 min at 37°C under a 5% CO_2_ atmosphere. After gently massaging the umbilical veins, the cell suspension was collected in a cell culture medium and supplemented with 1 mL fetal calf serum (FCS, Gibco) to inactivate collagenase. After two washes (350 ***g***, 12 min, 20°C), cells were resuspended in complete endothelial cell growth medium (ECGM, PromoCell, supplemented with 10% FCS), plated in 25 cm^2^ tissue plastic culture flasks (Greiner) and maintained at 37°C under a 5% CO_2_ atmosphere. BUVECs were cultured in modified ECGM medium [ECGM, diluted at 30% in M199 medium, supplemented with 5% FCS (Greiner) and 1% penicillin and streptomycin] with changing of medium every 2–3 days. BUVEC cell layers were used for infection after three passages *in vitro*. All bovine primary endothelial cell sample experiments were conducted in accordance with the Institutional Ethics Commission of Justus Liebig University of Giessen (Germany), and in accordance with the current European Animal Welfare Legislation: ART13TFEU.

### *Eimeria bovis* oocyst production

The *E. bovis* strain H used in the current study was originally isolated in the field in northern Germany and maintained since then by passages in parasite-free Holstein Friesian male calves (Fiege *et al*., 1992). For oocyst production, calves (*n* = 3) were orally infected at the age of 10 weeks with 3 × 10^4^ sporulated *E. bovis* oocysts. Experimental infections were conducted in accordance with the Institutional Ethics Commission of the Justus Liebig University of Giessen, Germany (allowance no. JLU 589_AZ). Excreted oocysts were isolated from the feces beginning 18 days p.i. according to the method of Jackson (1964). Sporulation of oocysts was achieved by incubation in a 2% (w/v) potassium dichromate (Merck) solution at room temperature (RT). Sporulated oocysts were stored in this solution at 4°C until further use. Sporozoites were excysted from sporulated oocysts as previously described (Hermosilla *et al*., 2002). Free sporozoites were washed three times in phosphate-buffered saline (PBS), resuspended in complete Iscove's modified Dulbecco's medium (Gibco), and counted using a Neubauer haemocytometer as described elsewhere (Hermosilla *et al*., 2008*b*). Sporozoite viability was determined by the trypan blue exclusion test according to Lang *et al*. (2009).

### Host cell cultures and parasite infection

BUVECs were cultured in 25 cm^2^ tissue culture flasks (Nunc) at 37°C and 5% CO_2_ atmosphere until confluency and infected with 5 × 10^5^ freshly excysted sporozoites. Cell culture medium was changed 1 day after infection and thereafter every third day. Infection rates were determined at 1 day p.i. microscopically.

### Protein extraction

Proteins from infected and non-infected BUVECs were extracted by cell sonication (20 s, five times) in radioimmunoprecipitation assay (RIPA) buffer [50 mm Tris-HCl, pH 7.4; 1% NP-40; 0.5% sodium-deoxycholate; 0.1% sodium dodecyl sulphate (SDS); 150 mm NaCl; 2 mm ethylenediaminetetraacetic acid; 50 mm sodium fluoride; all from Roth] supplemented with a protease inhibitor cocktail (1:200, Sigma-Aldrich). Cell homogenates were centrifuged (10 000 ***g***, 10 min, 4°C) to sediment intact cells and nuclei. The RIPA buffer-soluble protein content of respective supernatants was quantified using a Coomassie Plus (Bradford) Assay Kit (Thermo Scientific) following the manufacturer's instructions.

### SDS-PAGE and immunoblotting

For immunoblotting, samples were supplemented with 6 m urea protein loading buffer. After boiling (95°C) for 5 min, proteins (60 *μ*g per slot) were separated in 12 or 15% polyacrylamide gels by electrophoresis (100 V, 1.5 h; *tetra* system, BioRad). Proteins were then transferred to polyvinylidene difluoride membranes (Millipore) (300 mA, 2 h at 4°C). Blots were blocked in 3% bovine serum albumin (BSA) in Tris-buffered saline (TBS) [50 mm Tris-Cl, pH 7.6; 150 mm NaCl containing 0.1% Tween (blocking solution); Sigma-Aldrich] for 1 h at RT and then incubated in primary antibodies (Table 1) diluted in the blocking solution (overnight, 4°C). Detection of vinculin was used as a loading control for sample normalization. Following three washings with TBS-Tween 0.1% buffer, blots were incubated in adequate secondary antibody (Table 1) solutions (diluted in the blocking solution, 30 min, RT). Following three further washings with TBS-Tween 0.1% buffer, signal detection was accomplished by using an enhanced chemiluminescence detection system (ECL^®^ plus kit, GE Healthcare) and recorded using a ChemoCam Imager (Intas Science Imaging). Protein sizes were controlled by a protein ladder (PageRuler Plus^®^ Prestained Protein Ladder ~10–250 kDa, Thermo Fisher Scientific). Protein band intensities were quantified by using the Fiji Gel Analyzer plugin.

**Table 1.** Primary and secondary antibodies used in the current study

Antigen	Company	Cat. number	Origin	Dilution
Primary antibodies				
Vinculin	Santa Cruz	sc-73614	Mouse	1:1000
Cyclin B1	Abcam	ab32053	Rabbit	1:3000
Cyclin E1	Abcam	ab133266	Rabbit	1:1000
p27-kip1	Abcam	Ab32034	Rabbit	1:5000
p57-kip2	Santa Cruz	sc-56341	Mouse	1:5000
H3K9me2	Abcam	ab1220	Mouse	1:1000
Fibrillarin	Abcam	ab218846	Rabbit	1:1000
*Eimeria bovis*	In-house	–	Bovine	1:100
Name	Company	Cat. number	Reactivity	Dilution
Secondary antibodies				
Goat anti-mouse immunoglobulin g (IgG) peroxidase conjugated	Pierce	31430	Mouse	1:40 000
Goat anti-rabbit IgG peroxidase conjugated	Pierce	31460	Rabbit	1:40 000
AlexaFluor594	Thermo Fisher	R37117	Rabbit	1:500
AlexaFluor594	Thermo Fisher	A11005	Mouse	1:500
FITC	Thermo Fisher	A24441	Bovine	1:500

### Immunofluorescence assays

Cell layers were fixed with paraformaldehyde (PFA; 4%, 15 min, RT), washed thrice with PBS and incubated in the blocking/permeabilization solution (PBS with 3% BSA, 0.1% Triton X-100; 1 h, RT). Thereafter, samples were incubated in primary antibodies (Table 1) diluted in the blocking/permeabilization solution (overnight, 4°C, in a humidified chamber). After three washings with PBS, samples were subjected to secondary antibody solutions (Table 1; 30 min at RT and darkness). Cell nuclei were labelled with 4′,6-diamidino-2-phenylindole (DAPI) being present in mounting medium (Fluoromount G, Thermo Fisher).

Senescence assay was carried out using two experimental approaches to detect the *β*-galactosidase activity in non-infected and *E. bovis-*infected cells seeded in 12 mm coverslips coated with fibronectin (1:200, Thermo Fisher). First, using the Senescence *β*-Galactosidase Staining Kit (Cell signalling), and second by a fluorescence-based technique using the CellEvent™ Senescence Green Flow Cytometry Assay Kit (Thermo Fisher), following the manufacturer's instructions.

### Image acquisition and analysis

Images were collected with a Zeiss Confocal LSM 710 equipped with a motorized *XY* stage and an oil 40× Plan-Apochromat objective (numerical aperture of 1.3 Oil DIC MC27). All samples were previously fixed (PFA 4% in PBS) and stained with specific antibodies. Three channels were recorded for signal detection: blue/DAPI/405-laser, AlexaFluor488/green/argon-laser and AlexaFluor594/red/HeNe-543 laser. Images were acquired with a digital camera controlled by Zeiss ZEN 2010 software. The trophozoite-related image acquisition was performed in one level for each colour channel. Larger structures, such as immature and mature macromeront were imaged by *z*-stack optical series with a step-size of 0.3–0.5 μm depth. The *z*-series are displayed as maximum *z*-projections, and gamma, brightness and contrast were adjusted (identically for compared image sets) using Fiji software (Schindelin *et al*., 2012).

### Nuclear area and nucleoli number estimation

Measurements of defined parameters (i.e. area, perimeter, count, etc.) were performed by taking advantage of macro programming language in free open source software FIJI/ImageJ (version 1.53c) (Schindelin *et al*., 2012) and ilastik (version 1.4.0b5) (Berg *et al*., 2019). First, the summative intensity of *z*-stack images of nuclei and nucleoli was projected to obtain single images with a Fiji macro (https://doi.org/10.5281/zenodo.3971634). In the second step, the projected images were used for segmentation with the built-in Autocontext (two-stage) workflow in ilastik. The Autocontext workflow results were then exported as ‘simple segmentation’ ‘.tiff’ files for further analysis in Fiji. The segmented ilastik output images were analysed in two steps: (a) identifying each nucleus (within each image with a specific Region-Of-Interest (ROI) ID, automatically (https://doi.org/10.5281/zenodo.3971654) and (b) counting nucleoli within each nuclear ROI (https://doi.org/10.5281/zenodo.3971660). Both macros, described in (a) and (b), acquire the count, area and perimeter of their target particles. A schematic workflow is shown in Supplementary Fig. S3.

### Flow cytometry-based analysis of cell cycle phases and senescence

Cellular DNA content was measured using the FxCycle Far^®^ red stain reagent (Invitrogen, F10348) according to the manufacturer's instructions. The samples were analysed with a BD LSRFortessa™ cell analyzer (Becton-Dickinson, Heidelberg, Germany) applying 633/5 nm excitation and emission collected in a 660/20 band-pass. Cells were gated according to their size and granularity. Exclusively morphologically intact cells were included in the analysis. The *β*-galactosidase activity was measured using the CellEvent™ Senescence Green Flow Cytometry Assay Kit (Thermo Fisher) following the manufacturer's instructions. Data analysis was performed by using FlowJo^®^ (version 10.5.0) flow cytometry analysis software (FlowJo LLC, Ashland, OR).

### Statistical analysis

All data were expressed as mean ± s.e.m. from three independent experiments. For cell number- and fluorescence-activated cell sorting (FACS)-based assays, one-way analysis of variance (nonparametric) with Kruskal–Wallis post-test was performed using GraphPad Prism^®^7 software applying a significance level of 5%. Comparisons between non-infected and infected cells at each time were made by unpaired *t*-test applying a significance level of 5%.

## Results

### *Eimeria bovis* infection induces fusion of host cell nucleoli

**Fig. 1.**
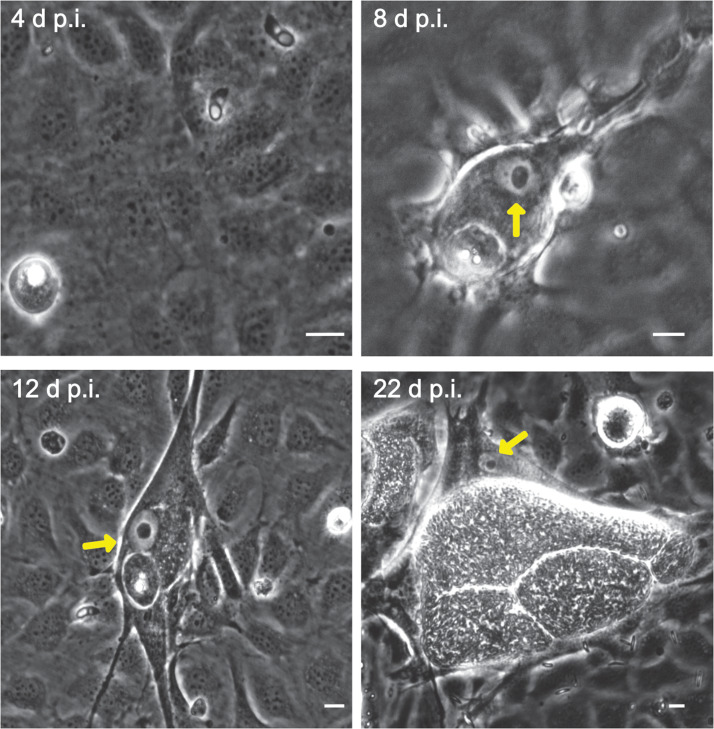
*Eimeria bovis in vitro* development in BUVECs. Three BUVEC isolates were infected with *E. bovis* sporozoites and monitored for further development by phase-contrast microscopy. Confluent BUVECs were infected with freshly isolated sporozoites and analysed from 4 to 22 days p.i. The cells were kept under controlled conditions of humidity, CO_2_ and temperature, and the same plate was followed in pictures over time. At 4 days p.i., intracellular sporozoites are detected with some of them developing into trophozoites. During 8–22 days p.i., early mature macromeront structures were observed. Starting with 8 days p.i., the fried-egg phenotype of the host cell nucleus is visible (arrows at 8, 12 and 22 days p.i.). The scale bar represents 10 *μ*m.

**Fig. 2.**
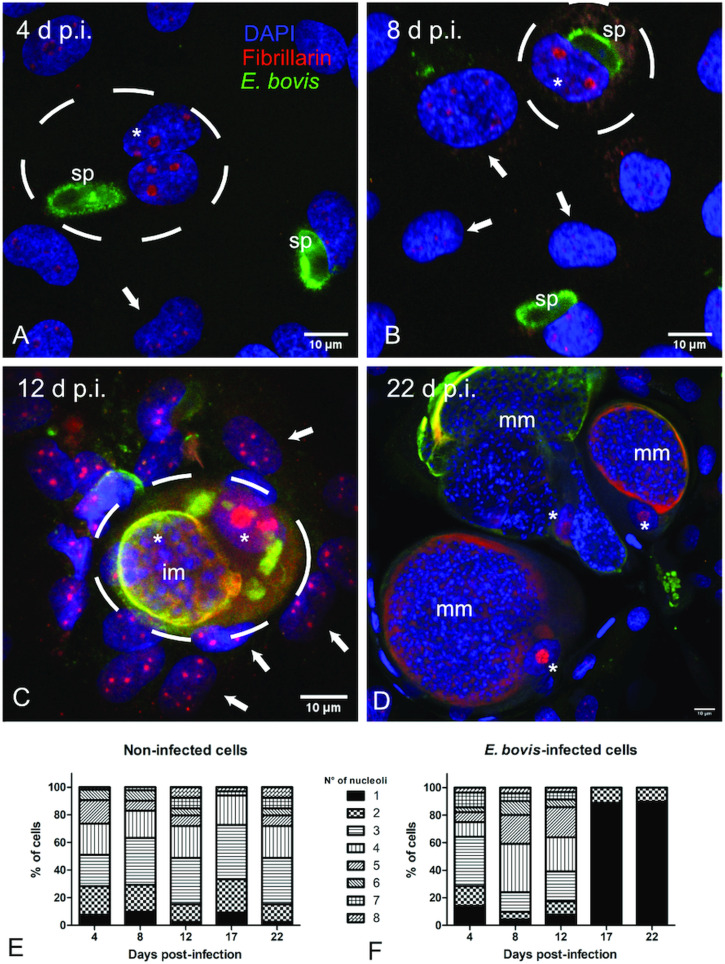
Fibrillarin-based nucleoli detection in *E. bovis-*infected BUVECs. (A–D) An exemplary illustration of host cell nucleoli in *E. bovis-*infected cells. Infected and non-infected BUVECs (*n* = 3) were stained by fibrillarin (red), anti-*E. bovis* hyperimmune serum (green) and DAPI (blue) to detect host cell nucleoli, parasite stages and nuclei, respectively. Although nuclei of non-infected cells showed a multinucleolar phenotype over time (indicated by white arrows), nuclei of *E. bovis*-infected cells revealed a continuous reduction in nucleoli numbers (indicated by asterisks) but an increase in size. Confocal images were acquired with 40× magnification. sp: sporozoite, im: immature macromeront, mm: mature meront. A 3D reconstruction of a meront-infected BUVECs is shown in a Supplementary video. (E) Quantification of the number of nucleoli per cell in non-infected and *E. bovis-*infected cells from 4 to 22 days p.i. The total number of non-infected and *E. bovis-*infected cells being included in the analysis was 849 and 565, respectively. The results represent the percentage of the nucleoli per cell in comparison with the total number of cells analysed at each time point. White dotted circles enclose the infected cell boundary. The scale bar represents 10 *μ*m.

**Fig. 3.**
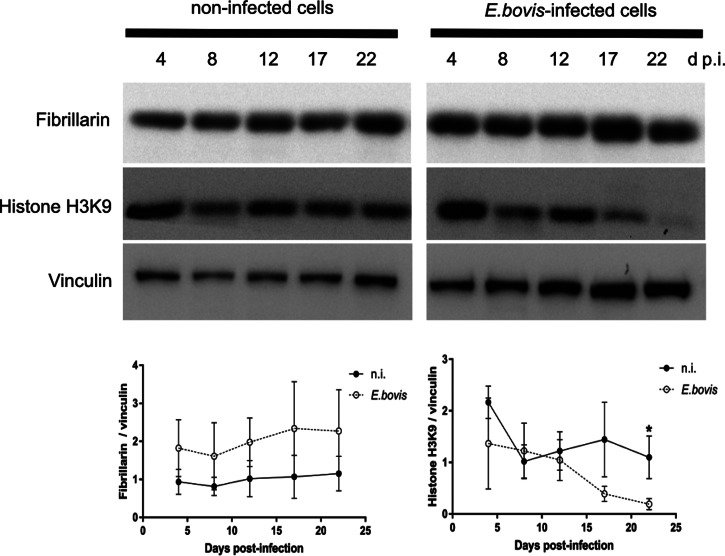
Fibrillarin and histone H3K9me2 abundance in *E. bovis-*infected BUVECs. Analysis of fibrillarin and HH3K9me2 expression in both non-infected and *E. bovis-*infected cells. Protein extracts from non-infected and *E. bovis*-infected BUVEC layers (three biological replicates) were subjected to western blotting and analysed for fibrillarin, histone HH3K9me2 (heterochromatin) and vinculin (loading control) expression. The density of the protein signals was quantified and plotted as a ratio relative to vinculin. The expression of HH3K9me2 was significantly reduced after 17 days p.i. in *E. bovis-*infected cells. No changes were for fibrillarin expression in all time points of parasite development when being compared with non-infected cells. Bars represent mean ± s.e.m. *P ≤ 0.05.

Microscopic monitoring of different developmental *E. bovis* stages during first merogony (trophozoites, immature meronts and mature macromeronts) *in vitro* confirmed a progressive and enduring change in a host cell nuclear phenotype from initially spotted, multi-nucleolar (at trophozoite and early immature meront stage, 1–7 days p.i. and also constantly present in non-infected host cells, see Supplementary Fig. S1) to fried-egg morphology (at immature to mature meront stage, from 8 days p.i. onwards, Fig. 1, yellow arrows) showing only one to a few enlarged nucleoli (according to Solovei *et al*. (2009), a normal bovine cell tends to contain more than three nucleoli per nucleus, which are homogeneously spread over the nuclear space). Similarly, fibrillarin-based staining indicated a decrease in nucleoli numbers in *E. bovis*-infected BUVECs over time (Fig. 2 and Supplementary video). Nucleolar enumeration confirmed the visual observation and revealed a progressive shift of nucleoli numbers towards 1–2 specimens/nucleus in mature macromeront-carrying host cells (Fig. 2E and F). Overall, almost 90% of infected cells showed only one nucleolus per nucleus at late merogony (Fig. 2F – the black proportion of the bars) whereas this phenotype was not at all detected in control cells (Fig. 2E). However, even though fibrillarin abundance seemed to be continuously enhanced in *E. bovis*-infected cell layers in comparison with non-infected control cells (as detected by using western blotting, Fig. 3), no statistical significance could be detected for this phenomenon (*E. bovis*-infected BUVECs *vs* control cells; *P* = 0.1750, Fig. 3) thereby indicating that *E. bovis*-driven effects mainly relied on alteration of nucleoli distribution within the nucleoplasm, most likely driven by nucleolar fusion or coalescence. Of note, a diminished number of enlarged nucleoli/nuclei is also reported for senescent cells (Mehta *et al*., 2007; Hein *et al*., 2012).

### *Eimeria bovis* infection alters heterochromatin distribution and expression in host cell nuclei

**Fig. 4.**
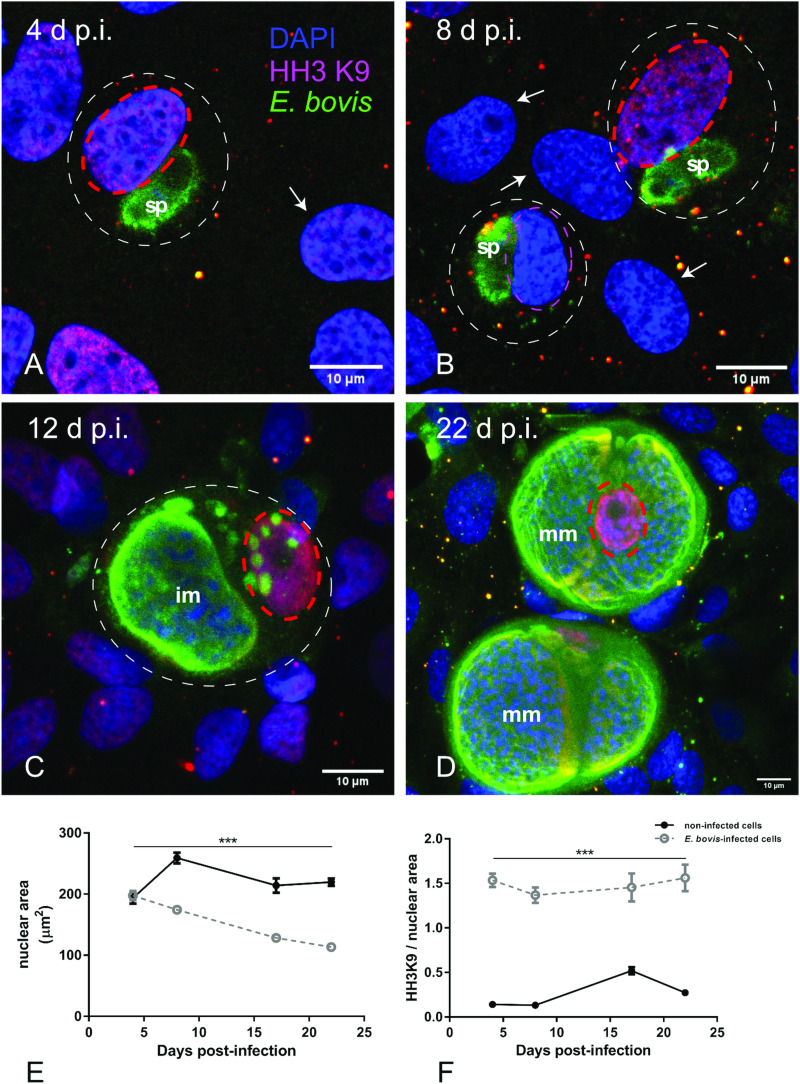
Histone H3K9me2 nuclear distribution in *E. bovis-*infected BUVECs. *Eimeria bovis-*infected and non-infected BUVECs (*n* = 3) were stained for HH3K9me2 (red), anti-*E. bovis* hyperimmune serum (green) and DAPI (blue) to detect heterochromatin, parasite stages and nuclei, respectively. The signal of HH3K9me2 (pink circles) increased from 4 days p.i. onwards in *E. bovis*-infected cells (A, B; white circles), leading to intense and homogeneous signals in macromeront-carrying BUVECs at 12 and 22 days p.i. (C, D) whereas non-infected bystander cells within the same monolayer showed hardly any signal for HH3K9me2 (white arrows); (E) estimation of the nuclear area of single non-infected and *E. bovis-*infected cells from 4 to 22 days p.i. showed that *E. bovis-*infected cells reduced progressively the host cell nuclei over time of infection whereas this was not altered in the non-infected monolayer. (F) The ratio of heterochromatin signal and the nuclear area in single non-infected and *E. bovis-*infected cells from 4 to 22 days p.i. showed a higher intensity of HH3K9me2 in the nuclei of the infected cells. However, almost no signal for histone H3K9me2 was detected in non-infected cells surrounding the infected host cell (white arrows) signal. Confocal images were acquired with 40× magnification. sp: sporozoite, im: immature macromeront, mm: mature meront. The scale bar represents 10 *μ*m. Bars represent mean ± s.e.m. *P < 0.0001.

To distinguish gene transcription-inactive heterochromatin from gene transcription-active euchromatin, we used typical markers detecting posttranslational modifications of core histones. Thus, histone 3 (H3) methylation at lysine 9 (K9) (H3K9me) is characteristic for heterochromatin, whereas H3 methylation at lysine 4 (H3K4me) is a marker for euchromatin (Yan and Boyd, 2006). Given that *E. bovis* development takes more than 3 weeks *in vitro*, fully confluent cells were always present in both, infected and control cultures. Consequently, a low to absent cellular duplication rate linked to low euchromatin abundance was expected. In line, we could hardly detect HH3K4me signals in these cultures, even though they were present in out-growing BUVECs (data not shown). Referring to HH3K9me2 signals, a homogeneous distribution of moderate heterochromatin signals in the nuclear compartment was observed during early merogony (4–8 days p.i.) in single *E. bovis-*infected cells (Fig. 4A and B and Supplementary Fig. S2). With ongoing macromeront growth and differentiation, the brightness and intensity of HH3K9me2-derived signals increased in meront-carrying BUVECs thereby suggesting enhanced heterochromatin formation (Fig. 4C and D). This reaction pattern remained stable until mature *E. bovis* macromeronts were fully formed (Fig. 4D, dotted red circle) and merozoites I were released. As an interesting finding, under identical image acquisition conditions, non-infected bystander cells within *E. bovis*-infected BUVEC layers that were directly surrounding infected cells showed low HH3K9me2 signals (Fig. 4A and B, white arrows) whereas non-infected cells in non-infected cell layers that were cultivated for the same time period as infected cell layers for control reasons revealed moderate but homogeneous signals for HH3K9me2 (see Supplementary data, S2). To correlate the nuclear HH3K9me2 signals of each nucleus with its individual size, a DAPI-based estimation of the single nuclear area was performed. Here we found a progressive diminishment of nuclear sizes in *E. bovis*-infected BUVECs during first merogony from 8 days p.i. onwards (*E. bovis*-infected BUVECs *vs* control cells: 4–22 days p.i.; all *P*s ≤ 0.0001; Fig. 4E). Overall, at 22 days p.i., the nuclei of macromeront-carrying BUVECs were up to 47% smaller than those of control cells (Fig. 4E). When calculating the ratio of HH3K9me2 signals to nuclear area, we could indeed confirm a significantly increased abundance of heterochromatin at single nucleus level for *E. bovis*-infected BUVECs during first merogony (*E. bovis*-infected BUVECs *vs* control cells: 4–22 days p.i.; all *P*s ≤ 0.0001; Fig. 4F). However, when analysing total HH3K9me2 protein expression in total cell layers, we found a decreased abundance of this molecule at late merogony (*E. bovis*-infected BUVECs *vs* control cells: 22 days p.i.; *P* = 0.0417; Fig. 3). This discrepancy may be related to the considerable decrease of the nuclear size in *E. bovis*-infected BUVECs and eventually also result from diminished heterochromatin presence in non-infected bystander cells.

### *Eimeria bovis* infection triggers host endothelial cell cycle arrest in G_1_ phase

**Fig. 5.**
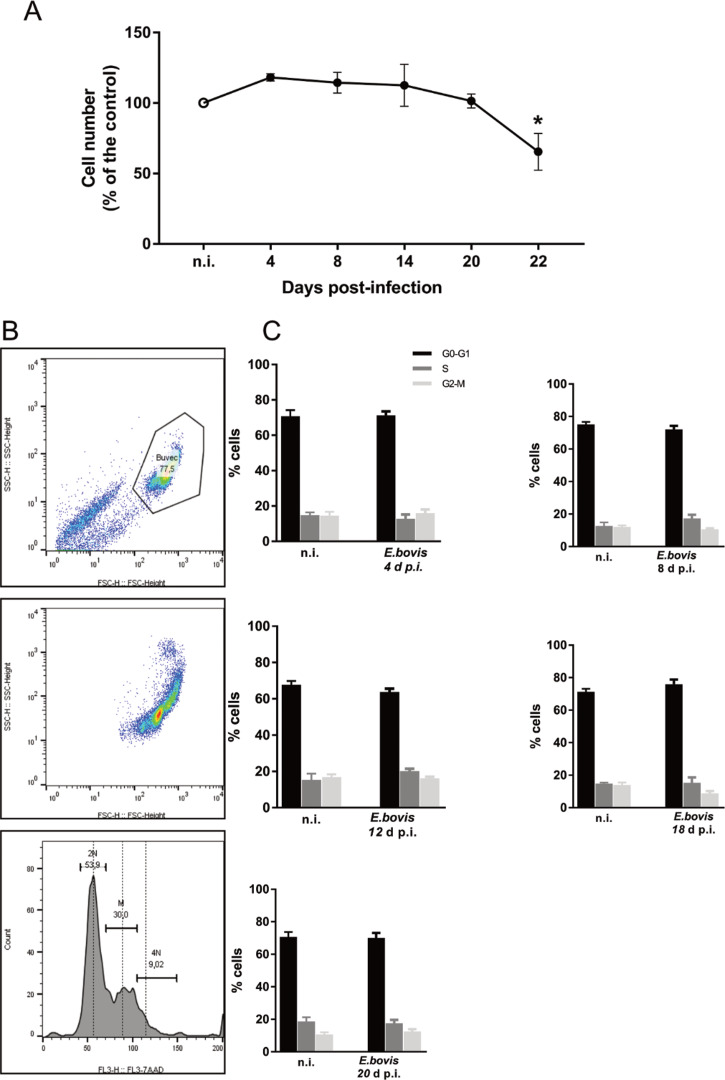
Estimation of cell numbers and cell cycle phases in *E. bovis*-infected BUVECs. (A) Confluent primary endothelial cells were infected with freshly isolated *E. bovis* sporozoites and analysed for cell numbers at 4, 8, 15, 20 and 22 days p.i. relative to non-infected controls (*n* = 3, each). The number of cells remains stable until 22 days when decreased by around 20%. Distribution of the cell cycle phases in *E. bovis-*infected BUVECs. Primary endothelial cells were infected with freshly isolated *E. bovis* sporozoites and the DNA content was examined at 4, 8, 12, 18 and 20 days p.i. (B) Exemplary illustration of a flowchart of the FACS-based analysis showing the total number of cells in G (one genomic DNA copy), G_2_ (two genomic copies) and S (the cell population in between both phases) phase. The cells were first gated to eliminate debris from the analysis. Furthermore, the DNA channel *vs* the population histogram was used to obtain the total number of cells in each peak. (C) Mean data obtained from *E. bovis*-infected cells (at 4, 8, 12, 18 and 20 days p.i.) were plotted as a percentage of the total cells *vs* DNA amount. Bars represent mean ± s.e.m. *P ≤ 0.001.

Effects of *E. bovis* infection on host cell proliferation were here examined using a simplistic approach by counting BUVEC numbers in control and *E. bovis*-infected cell layers. Given that *E. bovis* macromeront-carrying host cells increasingly rupture from 24 days p.i. onwards for merozoite type I release, which obviously will falsify cell enumeration, the experiments were restricted to 22 days of infection. Overall, *E. bovis* infections led to a significant decrease in host cell proliferation from 20 days p.i. onwards (*E. bovis*-infected BUVECs *vs* control cells: 22 days p.i.; *P* = 0.0004; Fig. 5A), leading to a reduction of total cell counts of 34.3% at 22 days p.i. Considering that the overall infection rate accounted for 21.4 ± 5%, this decrease of cell numbers could not be attributed to parasite-induced cell lysis even if host cell rupture would have occurred earlier than 24 days p.i. Thus, it seems likely that *E. bovis* infection blocks endothelial host cell proliferation towards macromeront maturation.

**Fig. 6.**
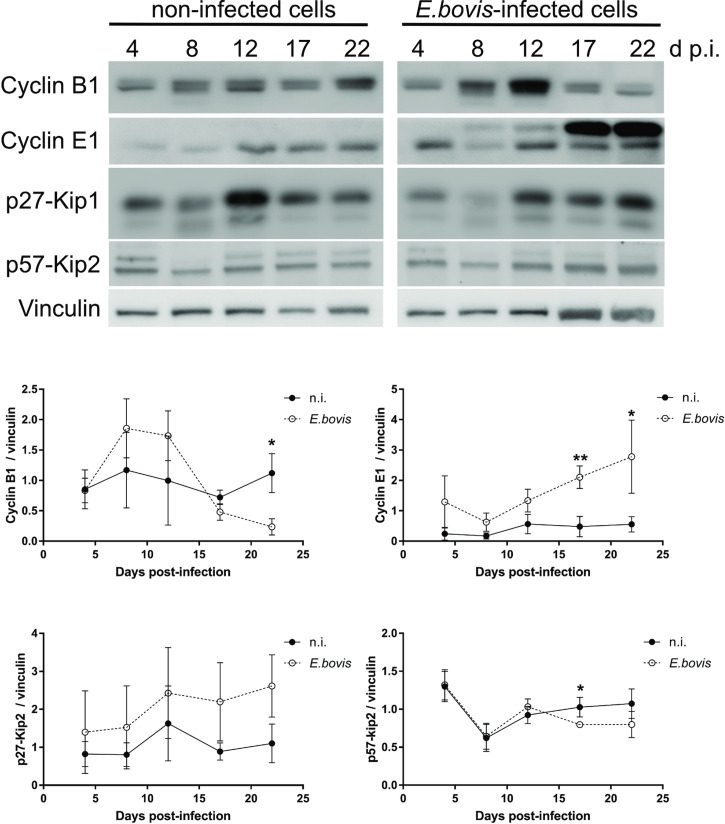
Cell cycle-related molecule expression in *E. bovis*-infected BUVECs. Three BUVEC isolates were infected with freshly isolated *E. bovis* sporozoites and analysed throughout *in vitro* infection (4–22 days p.i.) by western blotting for the abundance of the cell cycle-related molecules cyclin B1, cyclin E1, p27-kip1 and p57-kip2. Densities of protein signals were quantified and plotted as the ratio of the target molecule to vinculin (loading control). Cyclin B1, cyclin E1 and p57-kip2 were upregulated in infected cells thus indicating a G_1_ cell cycle arrest. Bars represent mean ± s.e.m. *P ≤ 0.05; ** *P* ≤ 0.005.

To verify whether *E. bovis* infections indeed interfered with host cell cycle progression, we performed FACS-based analyses on the different cell cycle phases by estimating cellular DNA content (Fig. 5B and C). This well-established method allows the discrimination of G_0_/G_1_, S and G_2_/M phases within the cell cycle. Overall, no significant differences were evident between *E. bovis*-infected BUVECs and control cells throughout merogony I (Fig. 5). Considering the fact that the typically low infection rate of 21.4 ± 5% may have masked parasite-driven effects when using this technique of rather low sensitivity, we additionally monitored the abundance of key molecules regulating G_0_/G_1_ and G_2_ phases. Western blot-based kinetic analyses revealed a diminished abundance of G_2_ phase-specific cyclin B1 at 22 days p.i. (*E. bovis*-infected BUVECs *vs* control cells; *P* = 0.0117; Fig. 6). Besides, a decreased p57-kip2 abundance was detected at 17 days p.i. (*E. bovis*-infected BUVECs *vs* control cells; *P* = 0.0394; Fig. 6). The most prominent reactions were attributed to G_1_ phase-specific cyclin E1 abundance, which continuously increased from 12 days p.i. onwards (*E. bovis*-infected BUVECs *vs* control cells; 17 days p.i.: *P* = 0.0048; 22 days p.i.: *P* = 0.0414; Fig. 6), thereby indicating that infected host cells were arrested in the G_1_ phase or at G_1_/S boundary. Interestingly, infected cells showed a fragmentation of cyclin E1 (from 8 days p.i. onwards) which was never observed in control cells (Fig. 6).

### *Eimeria bovis* infection induces senescence in host endothelial cell

**Fig. 7.**
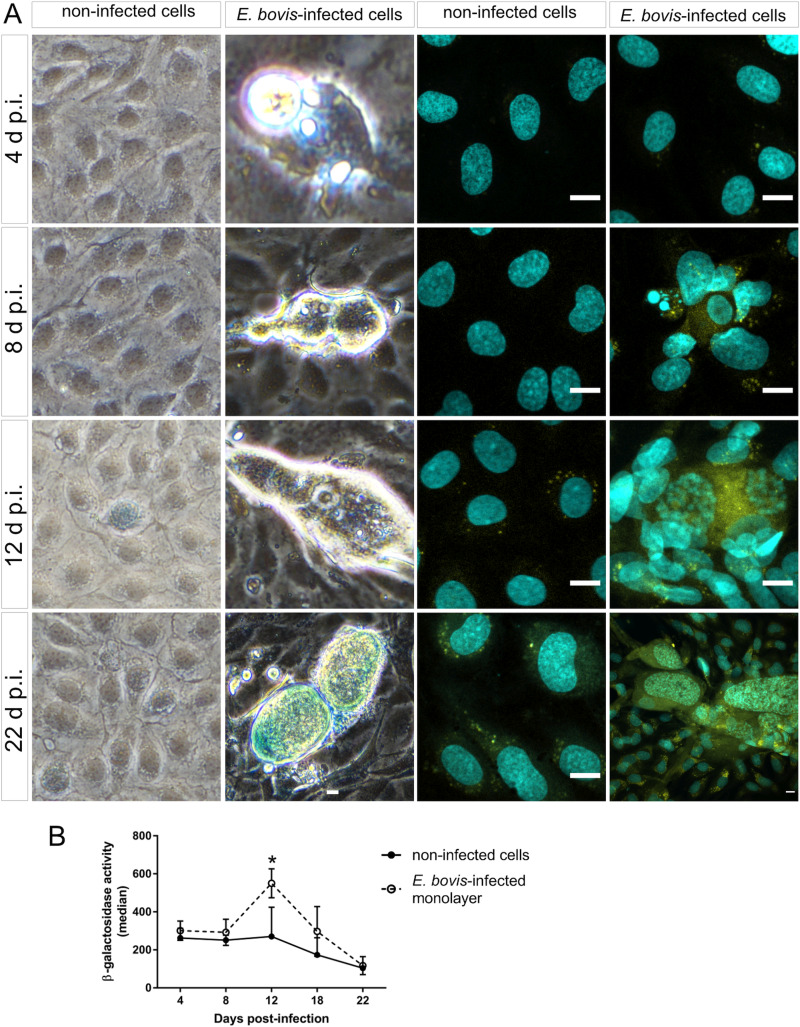
Beta-galactosidase activity in *E. bovis*-infected BUVECs. (A) *Eimeria bovis*-infected (4–22 days p.i.) and control BUVECs (both, *n* = 3) were stained for *β*-galactosidase activity by either a classical histochemical method leading to a blue precipitate in light microscopy (left panels of the figure) or to green fluorescence (right panels) as detected by confocal microscopy. By both techniques, an increase of the senescence activation from the first days of infection was observed. (B) Quantification of *β*-galactosidase activity in non-infected and *E. bovis-*infected BUVECs using FACS-based analysis show an increase in *β*-galactosidase activity in *E. bovis-*infected cells only at 12 days p.i. The scale bar represents 10 *μ*m. Bars represent the mean of fluorescence intensity ± s.e.m.; *P ≤ 0.05.

Cell cycle arrest at G_1_/S transition is a key feature of senescence, which is commonly induced in response to diverse cellular stresses (e.g. oxidative stress, starving and toxic compounds). One of the main hallmarks of senescence is the presence of senescence-associated *β*-galactosidase (SA-*β*-Gal) activity (Dimri *et al*., 1995; Lee *et al*., 2006; van Deursen, 2014). Using a classical enzyme histochemical staining and conventional phase-contrast microscopy, we demonstrated enhanced SA-*β*-Gal activity in almost all *E. bovis*-infected host cells irrespective of the days p.i. As such, *β*-Gal-positive signals were present around intracellular sporozoites at 4 days p.i. and also in immature and mature macromeront-carrying host cells between 18 and 22 days p.i. (Fig. 7A). However, it was difficult to illustrate this effect due to the large size and height of macromeronts (300 × 100 *μ*m) resulting in microscopic reflections. Therefore, we additionally carried out a fluorescence-based detection method for *β*-Gal quantification (Fig. 7A and B). The *z*-projections of single confocal images on total immature and mature *E. bovis* macromeronts confirmed enhanced SA-*β*-Gal activity in infected BUVECs (Fig. 7A). Similarly, FACS-based quantification revealed enhanced *β*-Gal activities at 12 and 17 days p.i. in *E. bovis*-infected host cells (Fig. 7B, and respectively control cells are shown in Fig. 7A); however, statistical significance was only evident in the case of 12 days p.i. (*E. bovis*-infected BUVECs *vs* control cells; *P* = 0.0479).

## Discussion

It is well-known that apicomplexan parasites considerably influence several intracellular pathways of infected host cells. Most of them are related to host cell control to improve parasite intracellular development, e.g. by modulating apoptosis, cholesterol/carbohydrate metabolism, host cell defense, cytoskeleton arrangement or the cell cycle (Brunet *et al*., 2008; Molestina *et al*., 2008; Hamid *et al*., 2014, 2015; Taubert *et al*., 2016; Silva *et al*., 2019). Similarly, *E. bovis* also depends on a live and healthy host cell to accomplish its massive proliferation during macromeront formation and has recently been shown to alter host cellular apoptosis (Lang *et al*., 2009), innate cellular defense mechanisms (Hermosilla *et al*., 2015) and host cellular metabolism (Hamid *et al*., 2015, 2014). Here, we analysed the influence of *E. bovis* infections on host cell cycle progression and senescence status.

For *in vitro* cultures, BUVECs were chosen as host cells to be rather close to the *in vivo* situation thereby also avoiding false influences on cell cycle or division activities driven by cell immortalization or tumoral origin. *Eimeria bovis* first merogony was fully accomplished in BUVECs with the formation of mature macromeronts I releasing merozoites I from 24 days p.i. onwards. It is important to note that we here exclusively analysed host endothelial cell layers for up to 22 days p.i. to avoid false-negative effects driven by parasite-induced host cell lysis.

In line with Taubert *et al*. (2010), *E. bovis* infection led to a phenotypic change in host cell nuclear morphology from 8 days p.i. onwards from spotted to fried-egg appearance, which is also observed in other ruminant macromeront-forming *Eimeria* species (Ruiz *et al*., 2013; Silva *et al*., 2015; López-Osorio *et al*., 2019). Here, we showed that nuclear fried-egg morphology corresponds to a single or double, centrally located, enlarged nucleolus (representing ‘egg yolk’) being surrounded by a heterochromatin-rich periphery (representing ‘egg white’). Applying fibrillarin, we proved that the central structure corresponds to host cell nucleolus most probably having been formed by fusion of several smaller nucleoli. This nuclear phenotype was infection-dependent because it was never observed in control cells. Nucleolus enumeration confirmed these findings showing that nucleoli decreased in number over time and macromeront-carrying host cells hardly showed more than one nucleolus. Remarkably, profiling of fibrillarin abundance showed that this effect was rather due to an altered nucleoplasmic distribution than to an overall change in the expression level because total fibrillarin abundance was not significantly altered by *E. bovis* infection. Interestingly, similar nuclear phenotypic changes were reported for senescent cells which consistently experience nucleolar fusion leading to reduced nucleoli numbers per cell nucleus (Mehta *et al*., 2007; Dillinger *et al*., 2017). Consequently, we additionally analysed typical markers of senescence as cell cycle-related status in *E. bovis*-infected cells. So far, parasite-triggered alteration of the host cellular cell cycle is a well-known feature acting on different phases and molecules of the cell cycle in a parasite-specific and even host cell type-specific fashion (Bouzahzah *et al*., 2008; Brunet *et al*., 2008; Molestina *et al*., 2008; Wiens *et al*., 2014; Kim *et al*., 2016; Diallo *et al*., 2019; Velásquez *et al*., 2019). Thus, *L. amazonensis* interferes early in cell cycle by G_0_/G_1_ phase arrest (Kuzmenok *et al*., 2005), *Encephalitozoon* infections induce an accumulation of host cells in the S phase (Scanlon *et al*., 2000). In a cell type-specific manner, *T. gondii* was shown to induce a shift from G_0_/G_1_ through the S phase with the accumulation of host cells in the S phase at the G_2_/M boundary in human foreskin fibroblasts, to trigger host cellular arrest in the G_2_ phase in a human trophoblast cell line, in human dermal fibroblasts and in an L6 rat myoblast cell line (Brunet *et al*., 2008; Kim *et al*., 2016) or to mediate a G_2_/M arrest in primary bovine endothelial cells (Velásquez *et al*., 2019). Referring to Eimeriidae, the only available report indicated a G_0_/G_1_ arrest in *E. tenella*-infected host cells (Diallo *et al*., 2019). In the case of *Plasmodium* spp., *Plasmodium falciparum* infections affected mitosis and led to a lack of cell division in HepG2 cells (Hanson *et al.,* 2015) but cell cycle-dependent reactions neither played a role in *Plasmodium berghei*- or *Plasmodium yoelii*-infected mouse models nor in *P. falciparum*-infected primary hepatocytes. Overall, amongst these known examples, an induction of senescence by intracellular parasites has been exclusively described for *T. cruzi*, so far (Guimarães-Pinto *et al*., 2018).

Cellular senescence occurs in response to excessive intra- or extracellular stress (Campisi, 2013). Senescence transition is characterized by several morphological and cellular changes, such as increased cellular size, nucleolar fusion and multinucleated nuclei (Hernandez-Segura *et al*., 2018). Hallmarks of senescence include a cell cycle arrest at the G_1_/S transition, increased activity of lysosomal SA-*β*-Gal, chromatin rearrangement and formation of senescence-associated heterochromatin foci (SAHF) amongst others (Hernandez-Segura *et al*., 2018). Besides, a secretory phenotype of senescent cells was also described affecting tissue homoeostasis by secreting distinct cytokines, growth factors and proteases (Tominaga, 2015).

To analyse the influence of *E. bovis* infections on host cellular senescence, we here estimated cell proliferation, SA-*β*-Gal activity, heterochromatin transition and expression of cell cycle-related molecules. In the case of *T. cruzi*-induced host cellular senescence, a remarkable reduction of cell proliferation was reported (Guimarães-Pinto *et al*., 2018). Concerning cell proliferation effects, it has to be mentioned that *E. bovis* culture in BUVECs leading to merozoite I formation is long lasting and takes more than 3 weeks of *in vitro* culture. However, BUVECs are generally confluent after 5–7 days of culture thereby ceasing proliferation based on contact-dependent events. Thus, it is difficult to detect considerable *E. bovis*-driven effects on host cell growth at times of macromeront formation. Nevertheless, simple cell enumeration experiments during *E. bovis* merogony I revealed a significant decrease in BUVEC numbers (34.6% reduction in comparison with the non-infected conditions) thereby indeed indicating interference of *E. bovis* infection with the host cellular proliferation capacity. However, FACS-based analyses on *E. bovis*-infected cell layers failed to reveal changes in cell cycle phases. This may be based on (i) a relatively low infection rate (which is typical for *E. bovis*; current study: 21.4%) and (ii) the rather low sensitivity of mere cellular DNA quantification for cell cycle phase discrimination. In contrast, when analysing distinct cell cycle-related molecules, we here found convincing evidence of an *E. bovis*-triggered arrest in the G_1_ phase. The control of the G_1_ phase in mammalian cells is mainly performed by two cyclin families, cyclins D and E (Sherr, 1993; Barnum and O'Connell, 2014; Liu *et al*., 2017). Knock-out studies showed that G_1_ cyclins can execute overlapping functions and that at least one class of G_1_ cyclins must be present to allow cell proliferation (Geng *et al*., 2003; Parisi *et al*., 2003; Kozar *et al*., 2004). In the current study, we showed that cyclin E1 accumulated in macromeront-infected BUVECs at times when merozoites I are formed. As an interesting finding, two additional proteins were detected by cyclin E1-specific antibodies exclusively in infected cells at late phase of merogony. So far, the significance of these bands remains unclear. Of note, other functions of cyclin E1 compared to cell cycle progression include proliferation, oncogenesis and apoptosis, and the cleavage of the cyclin E1 protein, specifically at the N-region, has been suggested to function as the apoptosis activator (Mazumder *et al*., 2002). Because apoptosis was shown to be blocked in *E. bovis-*infected cells (Lang *et al*., 2009), it remains unclear whether these findings are indeed related. *Toxoplasma gondii* controls host cell cyclin E expression by the use of a MYR1-dependent effector protein, HCE1 (host cyclin E) which allows parasites to modulate E2F transcription factor target genes (Panas *et al*., 2019). Another apicomplexan parasite, *B. besnoiti* was observed to upregulate the host cell cyclin E1 expression arresting cells in the G_1_ phase (Velásquez *et al*., 2020). Cyclin E1 is maximally active at the G_1_-to-S phase to induce DNA replication and is degraded during S/G_2_M (Caldon *et al*., 2013), suggesting that *E. bovis-*infected cells are not able to exit from the G_1_ phase. More recently, high levels of cyclin E1 were associated with DNA damage as well as with a cellular malignancy status (Caldon *et al*., 2013; Aziz *et al*., 2019). In agreement with a G_1_ arrest, we also found a decreased abundance of cyclin B1, which is the main protein controlling G_2_/M phase, suggesting that *E. bovis-*infected cells are not able to proceed into the M phase. Overall, the current data on cell cycle-related molecules indicated a G_1_ arrest which is also characteristic for senescent cells.

During senescence, chromatin transition to heterochromatin-rich foci (SAHF) is a well-described characteristic that was also confirmed for *T. cruzi*-infected host cells (Guimarães-Pinto *et al*., 2018). In general, two forms of chromatins are known: condensed heterochromatin and diffuse euchromatin. Euchromatin contains transcriptionally active or competent genes, whereas heterochromatin represents a tightly packed form in which gene expression may be silenced. In the current study, we applied HH3K9me2 as a marker of heterochromatin, which was also described as a useful marker of senescence (Grewal and Rice, 2004; Campos and Reinberg, 2009; Chandra *et al*., 2012). Overall, at a single-cell level, the nuclei of *E. bovis*-infected BUVECs showed considerably enhanced HH3K9me2 signals concomitant with a decreased nuclear size in *E. bovis-*infected cells which may reflect the formation of SAHF thereby indicating an infection-induced change in heterochromatin transition. Interestingly, this feature was linked to parasite proliferation and occurred when merozoites I were formed. As an interesting finding we additionally observed a diminished heterochromatin content in bystander cells directly surrounding infected cells. The rationale for this finding remains unclear, but it may have contributed to the finding that when measuring total cell layers (infection rate ~11%), the total HH3K9me2 abundance was lower in infected BUVECs compared to controls. Interestingly, HH3K9me2 is described as a key player in repressing lineage-inappropriate genes and shielding them for activation by transcription factors (Becker *et al*., 2017), suggesting that *E. bovis* may intentionally use increased HH3K9me2 abundance in single-infected cells for selective gene silencing. Of note, *E. bovis* sporozoites are always situated in proximity to the host cell nucleus after host cell invasion. By this intracellular sporozoite localization and by modulating heterochromatin abundance, the parasites could control the expression of distinct genes to support parasitic intracellular development, e.g. by inhibiting host cell apoptosis as previously described for this parasite (Lang *et al*., 2009; Taubert *et al*., 2010). However, the delicate balance between heterochromatin epigenetic maintenance and cellular processes should be carefully controlled to avoid consequences for genome integrity. As such, a loss of large blocks of H3K9 di- and tri-methylation has been correlated with gene expression changes in cancer cells and was suggested to contribute to their phenotypic plasticity (Feinberg *et al*., 2016).

As a hallmark of senescence, we also detected an enhanced activity of SA-*β*-Gal in *E. bovis*-infected host cells, by both, classical histochemical detection of SA-*β*-Gal and fluorescence-based approaches. Although enhanced staining of *E. bovis*-infected cells was easily visible on the microscope, it was difficult to document this effect in images based on the thickness and typical reflectance of macromeront structures. We therefore took advantage of fluorescence-based assays and could clearly illustrate increased SA-*β*-Gal signals in macromeront-infected BUVECs. Similarly, enhanced SA-*β*-Gal activities in *E. bovis*-infected BUVECs were also confirmed by FACS-based quantification thereby supporting an infection-triggered shift of host cells into a senescence-like phenotype. Interestingly, we also observed an enhanced proportion of SA-*β*-Gal-positive bystander cells. However, this observation needs thorough verification. In line with current data, overexpression of SA-*β*-Gal activity was also found in *T. cruzi*-infected fibroblasts (Guimarães-Pinto *et al*., 2018). These authors also showed that *T. cruzi*-infected host cells additionally exhibited a senescence-related secretory phenotype by increased production of interleukin (IL)-6, tumour necrosis factor-*α*, IL-1*β*, monocyte chemoattractant protein-1, nitrogen oxide and reactive oxygen species (ROS). In line, we have recently been detected enhanced levels of ROS in *E. bovis*-infected BUVECs (Z. D. Velásquez, unpublished data) which may indicate *E. bovis*-driven senescence-associated secretory phenotype (SASP). However, further studies are needed to support this assumption.

In summary, current data show that *E. bovis* induces a senescence-like state of infected primary host endothelial cells. So far, the rationale for this phenomenon remains unclear. In the case of *T. cruzi*-driven senescence, the formation of a long-term reservoir of parasites serving as a source of infectious stages for resident and recruited cells (especially in the skin) was hypothesized (Guimarães-Pinto *et al*., 2018). However, senescence is an important cellular tool to stop proliferation of damaged or dysfunctional cells, e.g. to restrict potential progression into malignancy (Serrano *et al*., 1997). Thus, the current phenomenon may simply be based on the massive endogenous stress driven by parasite-triggered exhaustion of cellular nutrients and building blocks in addition to the stressful, considerable expansion of the host cell. Conversely, it may also be driven by the parasite to keep its host cell viable as long as possible to benefit from cellular sources. In contrast to apoptosis, senescence keeps cells fully alive with functioning organelles while maintaining potential influences on neighbouring cells through secreted soluble factors (Childs *et al*., 2014) which could also be of advantage for *E. bovis*-triggered control of bystander cells physically supporting macromeront formation.

## Supplementary material

For supplementary material accompanying this paper visit https://doi.org/10.1017/S0031182020002097. click here to view supplementary material

## Data

The data that support the findings of this study are openly available in Zenodo at http://zenodo.org/ as micro formats extractor (doi: 10.5281/zenodo.3971634), nuclei counter (doi: 10.5281/zenodo.3971654) and particles in ROIs counter macros (doi: 10.5281/zenodo.3971660).
