# Supplementary figures and images for: Eimeria bovis infections induce G1 cell cycle arrest and a senescence-like phenotype in endothelial host cells
Source: Parasitology. 2020 Oct 26;148(3):341–53. doi: 10.1017/S0031182020002097 (PMC7890351; doi:10.1017/S0031182020002097)

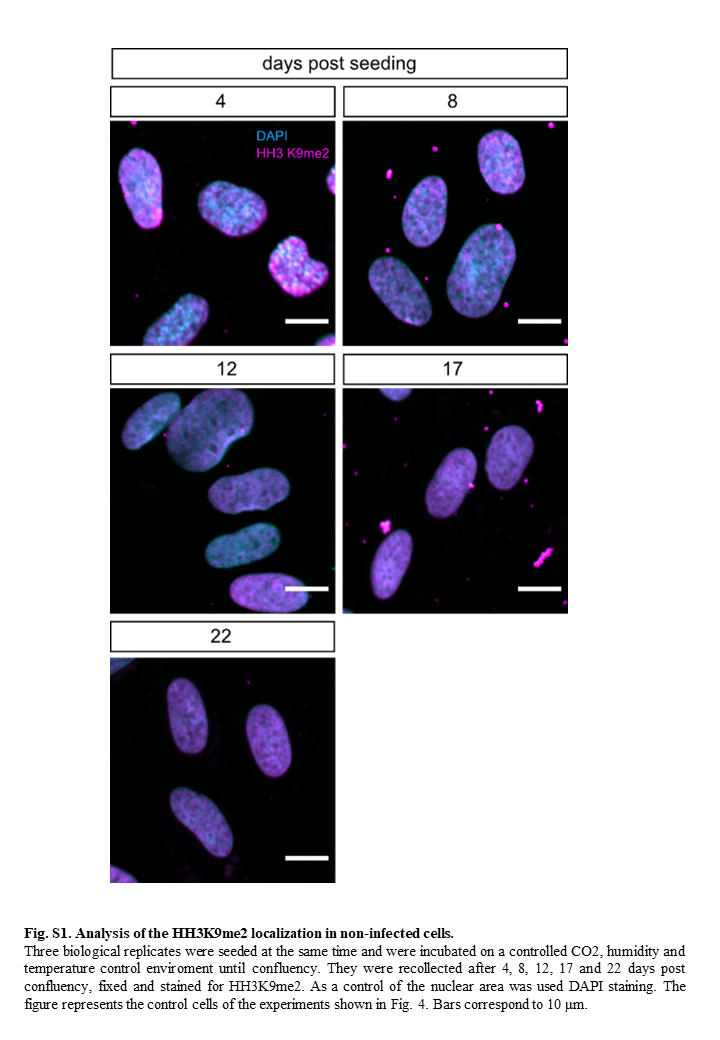

Supplement: Supplementary file 1 [file S0031182020002097sup001.zip › S0031182020002097sup003.tif]

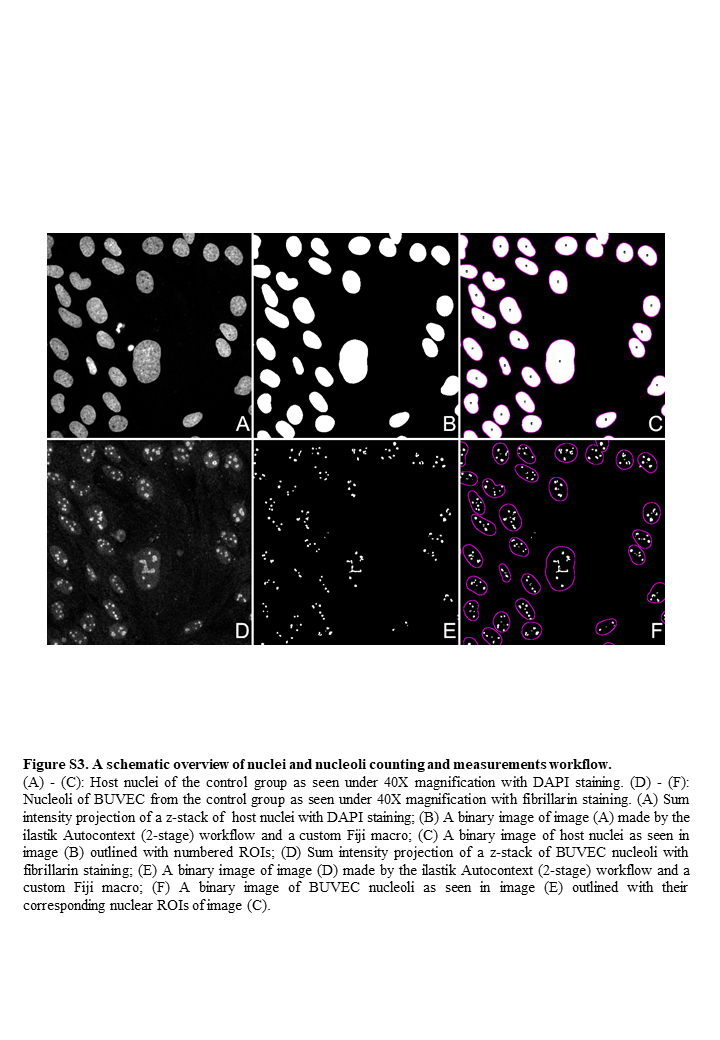

Supplement: Supplementary file 1 [file S0031182020002097sup001.zip › S0031182020002097sup005.tif]

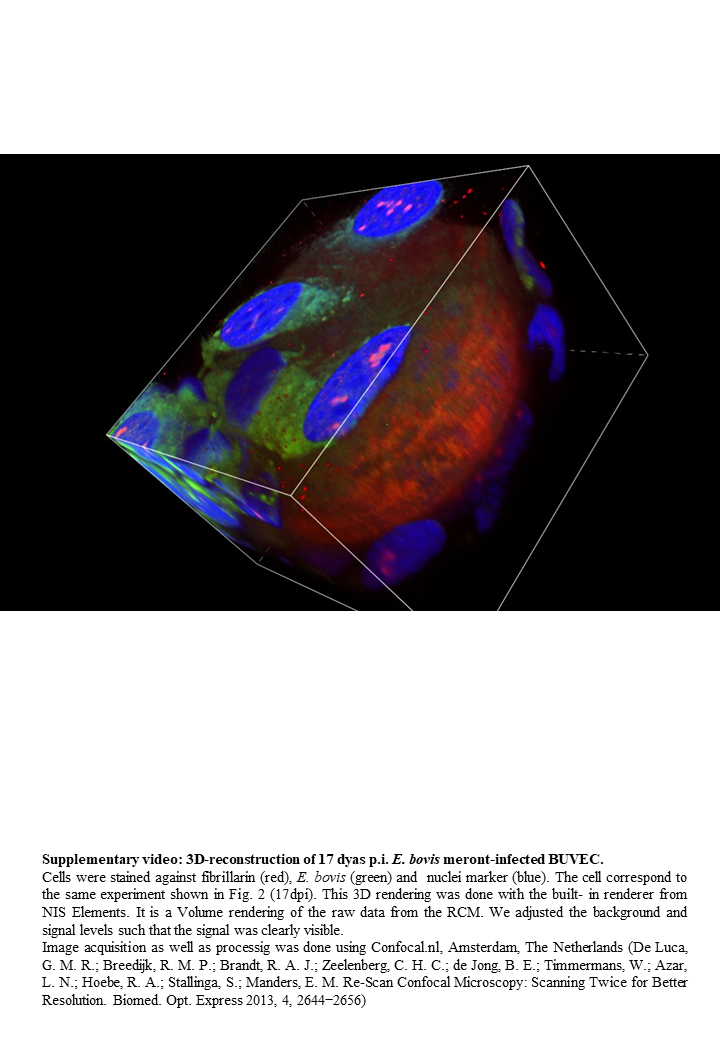

Supplement: Supplementary file 1 [file S0031182020002097sup001.zip › S0031182020002097sup001.tif]

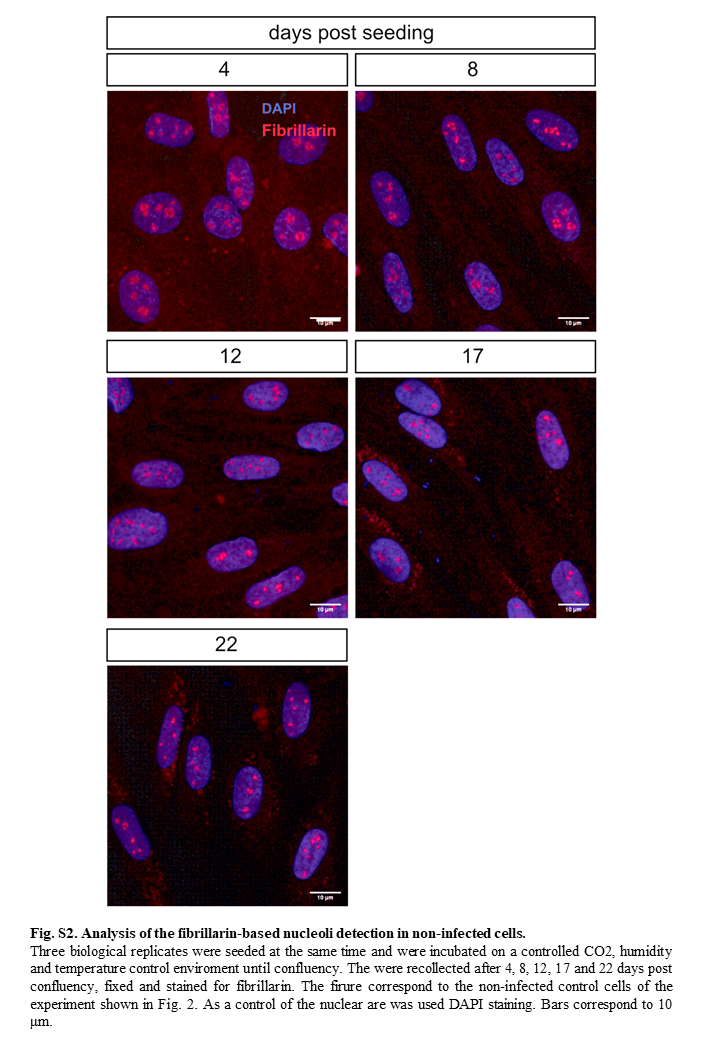

Supplement: Supplementary file 1 [file S0031182020002097sup001.zip › S0031182020002097sup004.tif]
